# Impact of Host IL28B rs12979860, rs8099917 in Interferon Responsiveness and Advanced Liver Disease in Chronic Genotype 3 Hepatitis C Patients

**DOI:** 10.1371/journal.pone.0099126

**Published:** 2014-06-10

**Authors:** Rushna Firdaus, Aritra Biswas, Kallol Saha, Anirban Mukherjee, Sujit Chaudhuri, Alok Chandra, Asokananda Konar, Provash Chandra Sadhukhan

**Affiliations:** 1 I.C.M.R. Virus Unit Kolkata, Indian Council of Medical Research, Kolkata, India; 2 Department of Gastroenterology, AMRI Hospitals Salt Lake, Kolkata, India; 3 Department of Medicine and Gastroenterology, Command Hospital, Kolkata, India; 4 Department of Gastroenterology, Peerless Hospital and B.K. Roy Research Centre, Kolkata, India; Rutgers, The State University of New Jersey, United States of America

## Abstract

**Background and Aims:**

Genetic polymorphisms near interleukin 28B gene are associated with spontaneous and treatment induced clearance of hepatitis C virus (HCV). Our objective was to evaluate the impact of interleukin 28B single nucleotide polymorphism (rs12979860, rs8099917) variability in HCV genotype 3 infected populations.

**Methods:**

400 hepatitis C seroreactive patients from different population groups in Eastern and North Eastern part of India were assessed for host and viral genotypic analysis. 83 HCV genotype 3 infected patients were administered pegylated interferon- ribavirin therapy. Viral genotyping was performed using nested reverse transcriptase-PCR followed by direct sequencing methods. Host interleukin 28B genotyping was performed using real-time PCR based single nucleotide polymorphism analysis.

**Results:**

Out of 400 hepatitis C seroreactive individuals, 73.25% were found to be RNA positive. HCV genotype 3 (65.87%) was found to be the major circulating strain in this region followed by genotype 1 (32.08%). rs12979860 CC genotype was significantly associated with sustained virological response in HCV genotype 3 infected population. In patients achieving rapid virological response, favourable CC/TT allele at rs12979860, rs8099917 was found to be predominant at both the alleles at 77%, 73.2% respectively; whereas in case of patients with relapsed HCV infection CT, TG alleles were found to be predominant. Additionally, CC genotypes at rs12979860 were found to be associated with sustained virological response in patients with high viral load (OR = 6.75, 0.05<p). HCV unfavourable rs12979860 TT, rs8099917 GG alleles were present in 34%, 27.6% patients with relapsed HCV infection respectively. Also unfavourable CT, TG genotypes were found to be predominant in patients with advanced stages of liver disease.

**Conclusion:**

CC, TT the two favourable markers at SNPs rs12979860 and rs8099917 are strongly associated with sustained virological response in genotype 3 infected populations. This information will aid clinicians to effectively design response based treatment regimen.

## Introduction

Chronic hepatitis C virus (HCV) infection is a major cause for developing cirrhosis and hepatocellular carcinoma (HCC), with an estimated global prevalence of 3% occurring in about 180 million carriers and more than 350,000 people die every year from hepatitis C related liver diseases [1], [2]. According to World Health Organization reports, HCV is found worldwide with certain countries having chronic infection rates as high as 5% or above. In India 12.5 million people are infected with HCV [2]. This virus is spread primarily by blood to blood contact associated with intravenous drug use, poorly sterilized medical equipment and improper transfusions [3]. Hepatitis C virus is a small (55–65 nm in size), enveloped, positive-sense single-stranded RNA virus of the family *Flaviviridae*. The genome consists of a single open reading frame of ∼9600 nucleotide bases long. This single open reading frame is translated to produce a single polyprotein, which is then further processed to produce 10 functional proteins. On the basis of genetic heterogeneity, the hepatitis C virus is classified into seven genotypes and further sub divided into several subtypes within each genotype [4]. The distribution pattern of HCV genotypes in patients with chronic hepatitis shows that in India; genotype 3 is the predominant strain followed by genotype 1 [5], [6]. Research also shows that within genotype 1; genotype 1b is less likely to develop viral drug resistance and therefore has a higher treatment cure rate than HCV genotype 1a [7]. Studies also show patients with genotype 1b were more likely to develop severe disease progression to chronic hepatitis C as compared to other genotypes [7]. Additionally, studies also observed that genotype 1 infected patients had a higher propensity of progressing to hepatocellular carcinoma than other genotypes [7]. However, some studies report that, infection with HCV genotype 3 was associated with faster fibrosis progression and higher degree of portal hypertension [8]. But till now, an increased incidence of HCC in cirrhotic patients infected with genotype 3 has not been documented. The gold standard therapy for chronic hepatitis C (CHC) consists of pegylated interferon (Peg-IFN) and ribavirin, but reports have shown the drugs are not well tolerated [9]. Therefore, it is the need of the hour to identify determinants of response to treatment.

Recent observations on the genetic determinants of CHC established that a single nucleotide polymorphism (SNP) in the interleukin (IL)-28B gene influences the treatment outcome of the HCV. IL28B gene encodes IFN-λ3, a cytokine, distantly related to type I IFNs and the interleukin (IL)-10 families. Together with IL28A (IFN-λ2) and IL29 (IFN-λ1), IL28B forms a cytokine gene cluster on a chromosomal region mapped to 19q13. Expression of the cytokines encoded by these three genes can be induced by ribonucleic acid (RNA) virus infection [10]. Studies have also shown that IL28B gene expression can inhibit HCV replication through Janus Kinase signal transducer and activator of transcription pathway in time and dose dependent manner [10]. In September 2009, Ge *et al.* [11] in a genome-wide association study (GWAS) found the rs12979860 single nucleotide polymorphism, which is located 3 kb upstream of the IL28B gene, to be the strongest host genetic predictor of SVR in hepatitis C genotype 1. They observed that rs12979860 CC patients, regardless of their ethnicity, reach sustained virological response (SVR) rates approximately twice than rs12979860 TT patients. The researchers also found that the highest percentage of favourable allele amongst Asians and lowest amongst Africans which in part explains the high rate of favourable response to interferon (IFN) in Asians as compared to Africans [11]. Another GWAS study in 2009 by Suppiah and Tanaka *et al* found that an additional polymorphism at rs80999917 which is located around 8 kb upstream of the IL28B to be a strong genetic determinant of SVR in HCV genotype 1 infected patients [11], [12], [13]

Different studies have shown that race, genotype, HCV RNA viral load, age, gender, basal metabolic index (BMI), fibrosis are important predictors for information regarding the IFN –ribavirin (IFN/RBV) treatment response [9]. The impact of IL28B associations in interferon responsiveness amongst Indian population remains understudied. In the current era, new HCV treatment paradigm includes one direct acting antiviral (DAA), a protease inhibitor (PI), in combination with Peg-IFN and ribavirin. The addition of DAA to Peg-IFN/RBV nearly doubles the chances of response to treatment but at the cost of increased toxicity [14]. These drugs have shown significant increase in SVR rates but the problem remains in the developing countries like India, where the DAA is still not introduced and if commenced; initial costs might be high for the people to afford the expensive treatment. Thus, in these clinical settings; IL28B genotyping in predicting IFN responsiveness would be beneficial for individualizing treatment approaches. The patients who carry favourable alleles might be eligible for shorter and cheaper regimens and inversely with unfavourable alleles would require longer therapy.

In the existing study, the host gene polymorphisms at rs12979860 and rs8099917 in the Indian cohort of patients in eastern and north-eastern part of India were investigated. Our study is probably the first attempt to correlate and understand the effects of the polymorphisms in eastern and north-eastern region of India.

### Objectives

The objectives of the study was to (i) compare the effects of IL28B polymorphisms in the progression of HCV infection at two different alleles at rs12979860 and rs8099917 with the prevalent genotype 3 strain circulating in India and (ii) to correlate the effects of the polymorphisms with interferon (IFN) response in different stages of liver diseases with other baseline predictors of response such as age, gender, basal viral load etc in HCV genotype 3 infected individuals.

## Methods

### Ethics Statement, patients and definition of treatment response

To participate in this study, informed consent was obtained for host and viral genetic analysis. This study protocol conformed to the ethical guidelines of the 1975 declaration of Helsinki and was approved by the ‘‘The Institutional Ethical Committee of National Institute of Cholera and Enteric Diseases, Indian Council of Medical Research’’.

A total of 400 HCV sero-reactive individuals were enrolled in this study from August, 2011 to July, 2013. Controls consisted [n = 100, male (n =  70) and female (n =  30)] of unrelated healthy subjects, who were negative for viral hepatitis markers and had normal serum levels of alanine aminotransferase (ALT), aspartate aminotransferase (AST). All the patients investigated were HBsAg and HIV negative. Both serum and whole blood was collected and stored at −80°C until further analysis.

Rapid virologic response (RVR) is defined as undetectable HCV RNA (<50 IU/ml) after 4 weeks of therapy; Sustained virological response (SVR) is defined as undetectable HCV RNA (<50 IU/ml) after 24 weeks end of therapy; Relapse is defined as undetectable HCV RNA at the end of therapy, but the reappearance of HCV RNA after end of therapy; Null Response (NR) is defined as less than a 2log-unit decrease in HCV RNA (IU/ml) from baseline after 12 weeks of therapy [15].

Liver function parameters like alanine aminotransferase (ALT), aspartate aminotransferase (AST), serum total bilirubin, creatinine was performed by kinetic rate methods (Beckman Coultier Synchron CX5Pro, USA).

### Host and Viral genotyping analysis

Genomic DNA was isolated from whole blood using the QIAamp DNA blood mini kit (Qiagen, Hilden, Germany) according to the manufacturer's instruction, genomic DNA concentration was measured by BioPhotometer spectrophotometer (Eppendorf, Hamburg, Germany).

Host SNP genotyping at rs12979860 C/T was performed by means of a TaqMan allelic discrimination assay. The primers and probes were used according to the methodology described by Ezzikouri *et al*, 2009 [16]. The probes were labeled with the fluorescent dye VIC and FAM respectively. The genotype for each sample was determined by the SDS 1.1 software for allelic discrimination (ABI 7500, Applied Biosystems, Foster City, CA, USA). The SNP rs8099917 were genotyped using a pre-designed TaqMan SNP genotyping Assay (Applied Biosystems; assay ID C_11710096_10). All the samples were repeated twice to check the results for quality control.

Viral RNA was extracted from HCV sero-reactive serum samples. Briefly, 140 µl of serum was used to isolate total viral RNA using QIAamp viral RNA mini kit (Qiagen, Hilden, Germany) according to the manufacturer's protocol and eluted with 50µl elution buffer. RNA was stored at −80°C until further use.

Detection of HCV viral RNA was done by nested RT-PCR based on 5′non-coding region (NCR) of HCV genome described elsewhere [17]. Briefly, first round RT-PCR was done in 20 µl total reaction volume containing 2 µl isolated RNA and second round nested PCR was performed in 25 µl total volume. A positive band at 256bp in 1.5% agarose gel stained with ethidium bromide was observed in gel documentation system (BioRad, USA) for HCV RNA positive samples.

Quantitative HCV RNA was estimated using in-house ABI real-time RT-PCR kit (AgPath-ID One Step RT-PCR kit). The HCV primers and probe sequences directed against the 5′ NCR of the HCV genome were designed in house [17].

Nested RT-PCR amplified amplicons of partial HCV core genome (405bp) was gel purified and directly used for DNA sequencing analysis in an automated DNA sequencer, 3130XL (ABI, USA) using Big Dye terminator 3.1 kit (Applied Biosystems, USA) [17]. The genotypes of the sequences obtained were determined using the NCBI genotyping tool [18].

### Statistical analysis

Continuous variables were presented as mean ± standard deviation or median (range) while categorical variables are expressed as frequencies (%). The existence of differences in groups was assessed by means of chi-square test for linear trend when appropriate and calculating the odds ratio (OR) within 95% confidence interval (CI). A p value ≤ 0.05 was used as the criterion for statistical significance. All the statistical analyses were performed using Statistical Package for Social Sciences (SPSS) program (version 10.0, SPSS Inc., Chicago, IL, USA).

## Results

### Patient's characteristics

**Figure 1:**
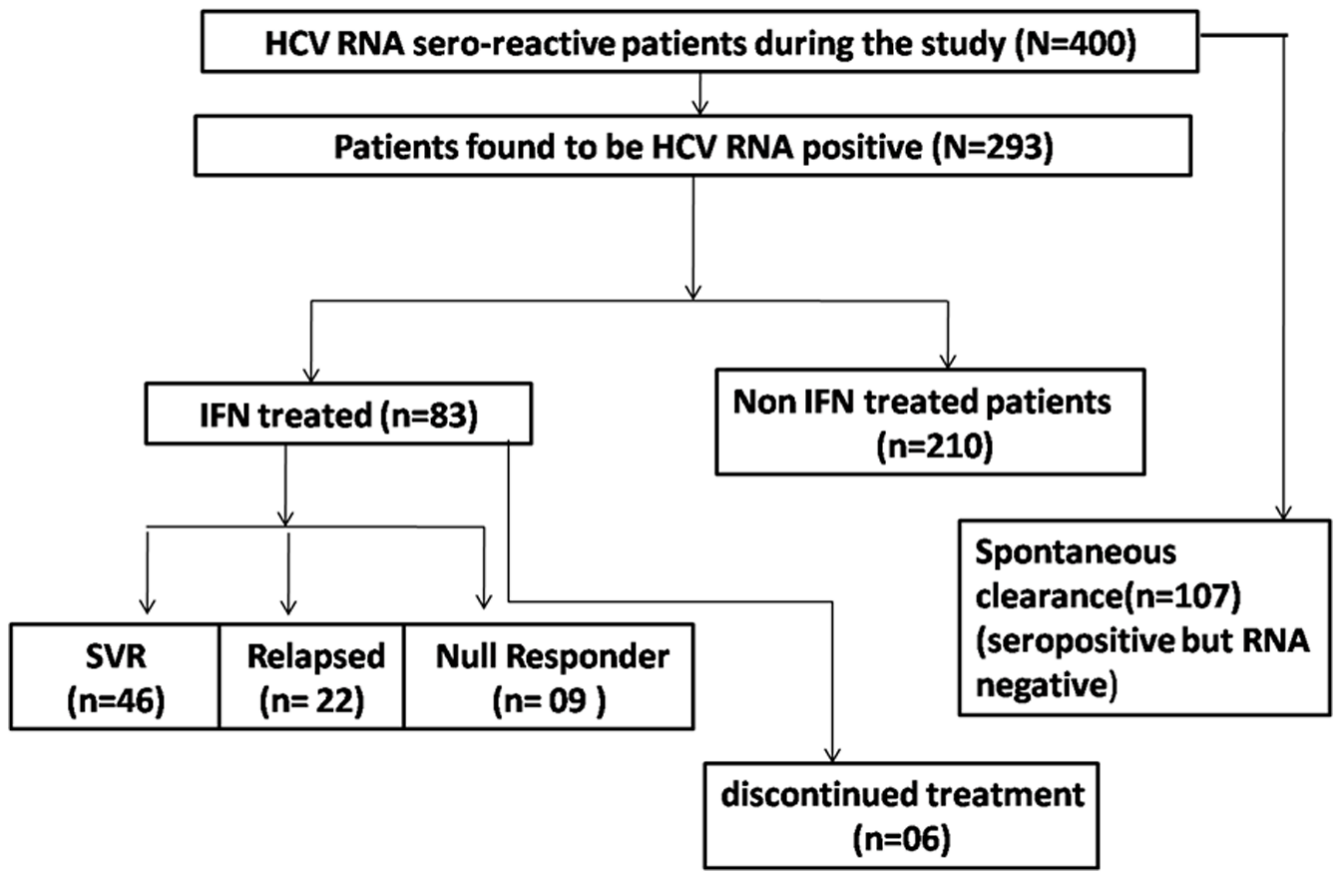
Flow chart depicting the samples sizes of the HCV patients enrolled in the study.

In this study, all 400 HCV sero-reactive patients were genotyped at SNP rs12979860, rs 8099917 for IL28B analysis. Out of the total, 293 (73.25%) were HCV RNA positive based on RNA quantitation (Figure 1). The demographic data, biochemical parameters, viral loads and viral genotypes of the samples assessed are summarized in Table 1. Among the study cohort males were 272 (68%) whilst females were 128 (32%). The mean age for females was 33.79±20.56 and for males were 31.05±18.91 years respectively. There was no statistical difference in the mean age between control and patient groups. ALT levels were significantly elevated in the patient groups compared to control population.

**Table 1:** Demographic and Baseline Clinical Characteristics of the Study Subjects Enrolled in the Study.

	HCV Sero-reactive Patients (n = 400)	Healthy Controls (n = 100)
**Mean age ± SD, years**	31.76±19.53	32.09±18.11
**Gender (%)**		
**Male**	68	62
**Female**	32	38
**Alanine Aminotransferase (IU/L)**	136.77±40.12	34.45±16.12
**Aspartate Aminotransferase(IU/L)**	129.65±25.31	36.15±12.22
**Mean billirubin (Total) (mg/dl)**	38.23±16.57	1.48±0.41
**Mean creatinine (mg/dl)**	120±62.23	1.28±0.21
**Viral genotypes (%)**	-	-
**Genotype 1**	31.44	-
**Genotype 3**	66.42	-
**Genotype 6**	2.14	-
**Patients With AdLD** 1	33	-

1 AdLD: Advanced liver disease (includes patients with liver cirrhosis)

Serum creatinine measured by alkaline picrate method, serum total billirubin was measured by diazo method, all enzymatic methods were done at 37°C, in a Beckman Coultier Synchron CX5 Pro, USA instrument

### HCV genotyping

Hepatitis C viral genotyping analysis revealed that genotype 3 (n = 193, 65.87%) was the predominant circulating strain followed by genotype 1 (n = 94, 32.08%) in this geographical region. Within genotype 3, HCV subtype 3a was the most commonly encountered strain accounting for 73.12% of the cases followed by genotype 3b in 26.88% individuals. Conversely, genotype 1b (n = 49, 52.12%) was the commonest within genotype 1 infected patients followed by genotype 1a (n = 43, 45.74%) and genotype 1c was found in only 2 (2.14%) individuals. A small percentage (n = 6, 2.05%) of new emerging genotype 6 was observed particularly in north-eastern isolates with history of intravenous drug uses (IVDUs).

### IL28B genotyping analysis

Host genotyping was done on 400 patients to determine the prevalence of the host genotypic markers at locus rs12979860 and rs8099917. The frequency of CC genotype associated with HCV clearance at rs1297960 was 70.75% (283 out of 400 individuals) compared to CT at 24% (96 out of 400). The distribution of genotype TT at locus rs12979860 reported to have poor HCV clearance rate was 5.25% (21 out of 400). Analysis of host genotype at rs8099917 reveals that the favourable genotype TT was present in 77.50% individuals (310 out of 400), TG was present in 15% individuals (60 out of 400), whereas GG, the unfavourable genotype was present in 7.50% individuals (30 out of 400).

Amongst the healthy subjects (n = 100), frequency of CC host genotype at locus rs12979860 was 73%, CT genotype was 23% whilst TT was 4%. Analysis of the allele frequencies shows that C allele frequency 0.85 whilst T allele frequency was at 0.15. The distribution of allelic frequencies was in accordance to the Hardy–Weinberg equilibrium (χ^2^  = 1.49, p<0.05). Similar analysis was performed for locus rs8099917 amongst healthy individuals showed that favourable allele TT was found in 70% individuals followed by TG allele at 30%; none of the individual reported GG at rs8099917. The allele frequencies at rs8099917 was also found to be in Hardy –Weinberg equilibrium with T allele frequency at 0.85 and G allele frequency at 0.15 (χ^2^ = 3.11, p<0.05).

**Figure 2:**
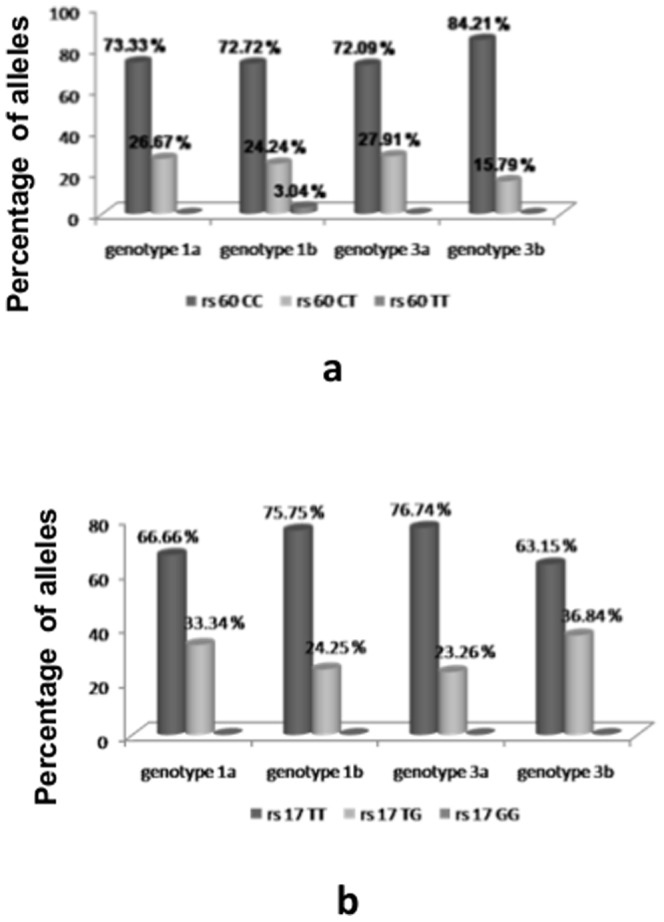
Frequency of (a) rs12979860 and (b) rs8099917 host genotypes in HCV genotype 3 infected within the study population.

Analysis of the host genotypes present within HCV genotype 3 infected individuals shows that CC allele at locus rs12979860 was predominant in both genotype 3a and genotype 3b at 72.09% and 84.21% respectively (Figure 2a). Similarly, at locus rs8099917 favourable TT allele was predominant in both genotype 3a and 3b at 76.74% and 63.15% respectively (Figure 2b).

### IL28B polymorphisms and response to IFN treatment

**Figure 3:**
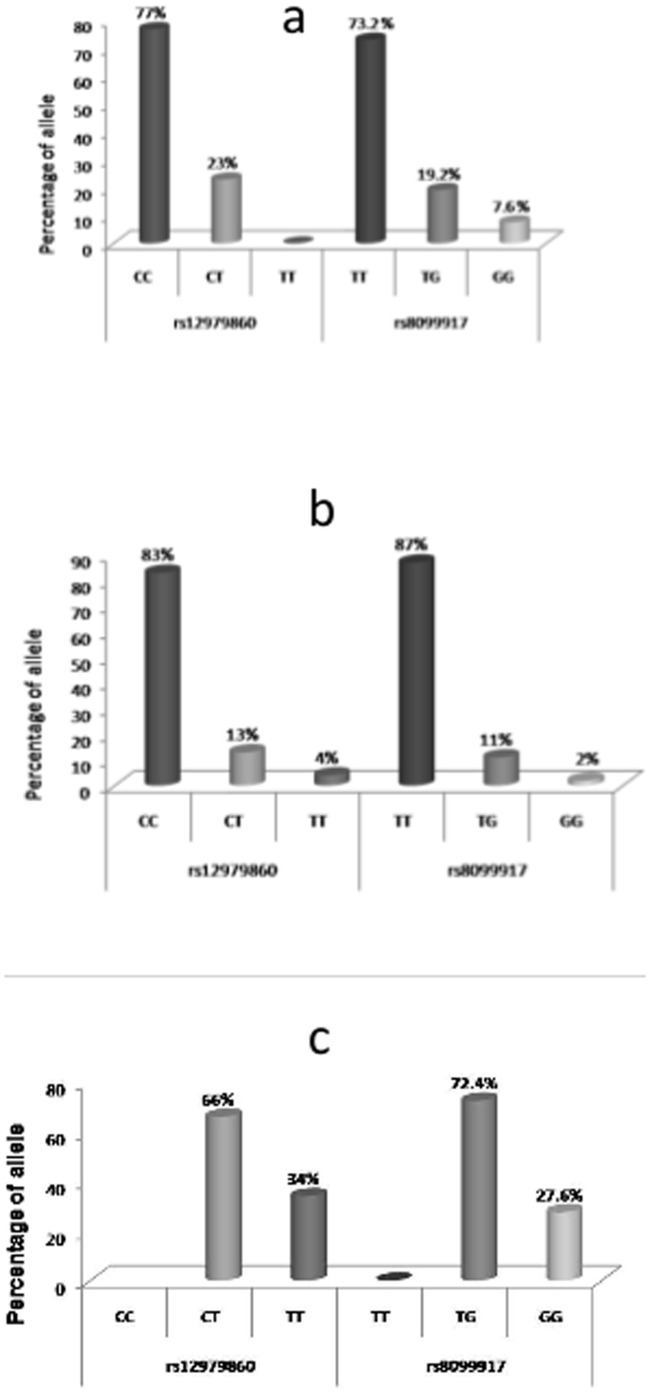
Frequency of rs129798960 host genotypes with (a) RVR, (b) SVR, and (c) Relapsed cases in Genotype 3 infected patients.

Among the 293 HCV RNA positive individuals, 83 genotype 3 infected individuals were treated with interferon and were evaluated for virological response (Figure 1). SVR was achieved in 46 out of 83 individuals (55.42%) while RVR was achieved in 26 individuals (31.3%) (Figure 1). In the interferon treated patient group, RVR was found to be an important predictor for SVR and can be considered as an important factor for determining the IFN response. Of the patients achieving RVR, 77% of them carried the favourable CC allele at rs12979860; whilst 73.2% of the individuals carried the favourable TT allele at rs8099917 locus (Figure 3a and 3b). In individuals with relapsed HCV infection, 66% of them carried CT allele at rs12979860 and 72.4% individuals carried TG allele at rs8099917 locus. Unfavourable TT and GG allele were present in 34% and 27.6% individuals at rs12979860 and rs8099917 loci respectively (Figure 3c).

We studied the influence of the IL28B genotype on the SVR in patients with either high or low baseline viral load for genotype 3 as rs12979860 is the single most statistically important predictor. Our results show that host genotype CC at rs12979860 was statistically correlated with high viral load but not with low viral load for genotype 3 infected individuals (Table 2).

**Table 2:** Influences of Host Genotypes of rs12979860 on SVR in High or Low Baseline Viral Load.

Baseline viral load	rs12979860	NON SVR N (%)	SVR N (%)	OR (95% CI)	p value
Low viral load (<0.5×10^6^ IU/ml)	CC	05	20	1.77 (0.3841 to 8.2282)	0.46
	CT/TT	09	04		
High viral load (≥0.5×10^6^IU/ml)	CC	03	13	6.74 (1.4905 to 30.4851)	0.013
	CT/TT	14	09		

OR: Odds ratio, CI: Confidence interval.

Analysis of treatment naive HCV infected individuals showed that rs12979860 CC allele was predominant with 146 out of 210 (69.50%) individuals carrying this favourable allele, followed by rs12979860 CT allele being present in 55 individuals (26.3%); unfavourable TT allele was present in 4.2% individuals. Similarly, favourable rs8099917 TT allele was present in 78.09% (164 out of 210) of treatment naive HCV infected patients, whereas rs8099917 TG allele was present in 14.28% individuals (30 out of 210) and unfavourable rs8099917 GG allele was present in only 7.63% individuals (15 out of 210). Thus in our study it was seen that favourable alleles were dominant within our study population.

### IL28B polymorphisms in advanced liver disease (AdLD)

**Figure 4:**
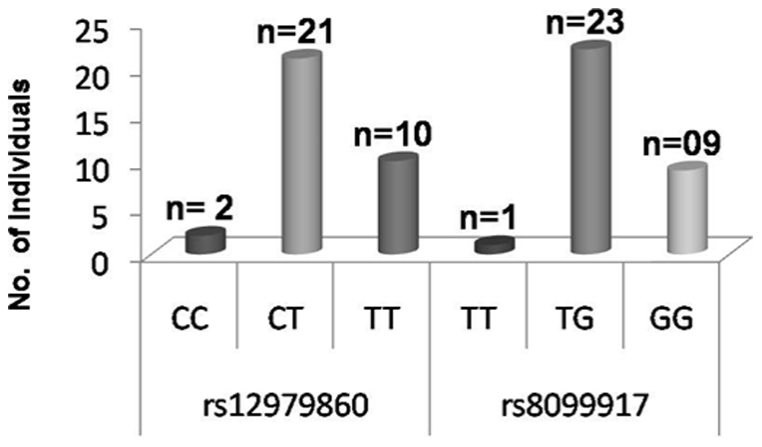
Frequency of IL28B host genotypes in HCV infected patients with (genotype 3) Advanced liver disease (AdLD) (N = 33).

Clinical observation showed that 33 out of the 293 HCV RNA positive individuals (11.2%) were diagnosed to be in different stages of advanced liver diseases (AdLD). Analysis of the frequencies of IL28B genotypes showed that within this group, heterozygous CT genotype (63.63%) was the predominant allele at rs12979860 and TG genotype (69.69%) at rs8099917 (Figure 4). Favourable genotype CC and TT at rs12979860, rs8099917 were found in only two and one individual respectively.

## Discussion

HCV usually induces a robust immune response but it has developed mechanisms to escape immune defense and establish persistent infection [8]. Recent reports indicate that 12.5 million people are infected in India and the number is steadily increasing day by day [5]. As per current guidelines for clinical practice, HCV genotype and HCV RNA levels at baseline are considered to be an important marker before starting the antiviral interferon therapy but studies show that the treatment progression is often marred by side effects which ultimately force the patients to discontinue the therapy [10].

Ge *et al*. (2009), found that rs12979860 single nucleotide polymorphism, located 3 kb upstream of the IL28B gene was the strongest host predictor for treatment response in HCV patients infected with genotype 1 strain [11]. Tanaka et al. (2009), found rs8099917, another IL28B polymorphism which is located around 8 kb upstream of the IL28B gene, to be the strongest genetic determinant of SVR in HCV genotype 1 infected patients [12]. Initially, most of the studies have concentrated on the HCV genotype 1 as this genotype is more common in western countries [19]. However, the impact of these polymorphisms within genotype 3 HCV infected individuals remains understudied. Reports have also indicated that ethnic differences in the IL28B gene polymorphisms may explain at least in part, the different outcome rates of HCV infection in different population groups [20], [21], [22].

Differences in treatment outcome with respect to antiviral treatment have led the researchers in the present study to ascertain the frequency of IL28B host genotype within different population groups in eastern and north eastern India. The Indian population is ethnically heterogeneous; therefore, genetic variability is expected. Not much data are available about the frequency of host genotype rs12979860 or rs8099917 in India, a study conducted by Sivaprasad *et al* (2012), amongst south Indian population demonstrated that the proportion of CC allele was higher than the proportion of CT, TT at rs12979860 alleles in the south Indian population, but the study concentrated only on the proportion of the host alleles amongst the healthy individuals [23]. Our study on the other hand analyzed both the healthy individuals as well as HCV infected individuals, to get a clear picture of the genetic disposition of the host genotypes. In this study, analysis of healthy individuals showed that our data is in concordance with the data obtained by Sivaprasad *et al* (2012) but the percentage of favourable CC allele at rs12979860 locus was higher at 73% in contrast to 59.09% [23]. Additionally, our study analyzed the host genetic disposition at both rs12979860 and rs8099917 amongst healthy individuals which was not done elsewhere.

The association between IL28B polymorphism and the progression of liver fibrosis is still controversial, as the possible underlying mechanisms are still unknown to us [24], [25]. Within our study population, viral genotype 3 was found to be most widely circulated strain; HCV genotype 3 is reportedly associated with steatosis and rapid progression to HCC. Due to its association with steatosis, we have studied the proportion of IL28B genotypes in HCV infected patients in different stages of advanced liver diseases (AdLD). We observed that the percentage of unfavourable CT and TG allele were more in these patients as compared to favourable CC and TT allele at rs12979860 and rs8099917 respectively (Figure 3).

Several reports indicate strong association between IL28B polymorphism and SVR; the mechanisms are still not properly understood. Response guided therapy for CHC patients based on IL28B genotypes, viral genotypes and treatment response is the current treatment strategy. RVR was found to be the useful predictor for SVR in treatment of CHC with peg-IFN plus ribavirin. With this aspect in mind, we examined the host genotypes associated with different IFN responses. In patients with RVR, CC genotypes at rs12979860 were present in 77% of the patients whereas the unfavourable CT was present in only 23% of the individuals. Interestingly, none of the patients achieving RVR reported unfavourable TT allele at rs12979860. Similarly, at locus rs8099917, response rates were 73.2% TT, 19.2% TG, followed by 7.6% GG allele (Figure 3a). Similar results were obtained in patients with SVR which shows that in 83% of the patients achieving SVR had the favourable CC allele at rs12979860. Interestingly, in patients with relapsed infection it was seen that 66% of the patients had unfavourable CT allele at their rs12979860 locus followed by 34% TT allele. Also at rs8099917, TG genotype was present in 72.4% individuals followed by GG host genotype in 27.6% individuals (Figure 3b). Therefore, it could be assumed that favourable genotypes at both rs12979860 and rs8099917 could be important markers for the prediction of the favourable results in patients undergoing IFN therapy in genotype 3 infected patients (Figure 3a and 3b). Within treatment naive HCV infected individuals, our results showed that the percentage of favourable allele rs12979860 CC and rs8099917 TT was higher; therefore it could be assumed that within our study population the propensity to towards favourable alleles is higher and it could be a valuable input for physicians in predicting the treatment response.

The limitation of the study was that the number of samples size with patients undergoing IFN treatment was small. A large cohort of patients undergoing IFN therapy is needed to explain in detail how the SNP rs12979860 and rs8099917 could influence the treatment response in HCV genotype 3 infected population.

Our results are the first report from this part of the country providing information about the impact of IL28B genotypes in different HCV infected population groups from eastern and north-eastern India. Our results for the first time indicate that for HCV genotype 3 infected patients, CC genotype at rs12979860 is an important predictor for patients to achieve RVR. Additionally our results also emphasized the point that rs12979860 CC host genotype was significantly associated with SVR in genotype 3 infected individuals with high viral load. To best of our knowledge, our data on SNP rs8099917 is the first Indian data within HCV infected population to be reported from this region.

In conclusion, it could be said that in the current scenario, where the cost of IFN treatment for HCV is very high to the people of our country where the healthcare system is already overburdened with lack of resources. Therefore, it is the need of the hour to shift our focus to how the treatment mechanisms can be tailor-made to suit every individual's genetic makeup.

## Ethical Approval

This study received approval from the institutional review boards in all participant sites.
